# Imaging Aspects in a Case of Persistent Müllerian Duct Syndrome (PMDS): A Case Report and Overview

**DOI:** 10.7759/cureus.65880

**Published:** 2024-07-31

**Authors:** Vlad-Octavian Bolocan, Georgian-Florentin Diaconu, Alexandra Giuvelea, Mihaela Secareanu, Cosmin Medar, Loredana S Manolescu, Amelia Petrescu, Viorel Jinga

**Affiliations:** 1 Department of Clinical Laboratory of Radiology and Medical Imaging, Clinical Hospital "Prof. Dr. Theodor Burghele", Bucharest, ROU; 2 Department of Fundamental Sciences, Faculty of Midwifery and Nursing, University of Medicine and Pharmacy "Carol Davila", Bucharest, ROU; 3 Department of Pathology, Clinical Hospital "Prof. Dr. Theodor Burghele", Bucharest, ROU; 4 Department of Urology, Clinical Hospital "Prof. Dr. Theodor Burghele", Bucharest, ROU; 5 Department of Urology, Faculty of Medicine, University of Medicine and Pharmacy "Carol Davila", Bucharest, ROU; 6 Medical Sciences Section, Academy of Romanian Scientists, Bucharest, ROU

**Keywords:** müllerian duct, pseudohermaphroditism, management, uterus, male

## Abstract

Persistent Müllerian duct syndrome (PMDS) is a rare kind of internal male pseudohermaphroditism. The patient, who has a male karyotype and phenotypic characteristics, exhibits Müllerian duct derivatives such as the uterus, cervix, fallopian tubes, and upper two-thirds of the vagina. This article provides a comprehensive analysis of the CT and MRI characteristics of a case of PMDS in a 35-year-old male patient who sought medical attention at our clinic due to pain in the left inguinal region and the presence of undescended testes on both sides. The imaging results showed a pelvic mass with a bicornuate appearance, situated adjacent to the bladder on the left side. The diagnosis of compensated hypergonadotropic hypogonadism with a normal male karyotype is confirmed through biological and genetic studies. The final diagnosis was confirmed through histopathological examination following laparoscopic transperitoneal surgical removal. The examination revealed a left lateral vesical pelvic tumor with a firm-elastic, bicornuate appearance, along with a thickened endometrium. Microscopic findings included simple glandular hyperplasia with edema in the endometrium, a small adenomatous polyp at the uterine fundus, and bilateral rigid cords consistent with vas deferens histology. The primary issue with PMDS is in its rarity, which consequently limits the availability of comprehensive case series and prospective research. As a result, radiologists and surgeons must possess knowledge of this ailment, as there is a scarcity of defined treatment guidelines and long-term care strategies.

## Introduction

Persistent Müllerian duct syndrome (PMDS) is a type of internal male pseudohermaphroditism (MPH) where the presence of the uterus and fallopian tubes indicates a failure in the sex differentiation pathway that relies on anti-Müllerian hormone (AMH) [1]. Consequently, these patients would have external genitalia and typical male traits, confirming the integrity of the androgen-dependent system. During imaging examinations for other problems or surgical correction of cryptorchidism or inguinal hernia, the Müllerian derivatives are typically detected inadvertently in PMDS. There have been two distinct anatomical forms that have been identified: In the typical scenario, one testis descends into the scrotum and pulls down the fallopian tube on the same side, as well as the uterus, and subsequently the fallopian tube and testis on the opposite side. Occasionally, both testes might be found in an ovarian position, where they are located within the wide ligament. PMDS is a genetic disorder that occurs when there is a deficiency in the synthesis of AMH or when the target organs are resistant to AMH [2]. When dealing with Müllerian duct anomalies, it must be taken into consideration that these are often associated with other congenital malformations, such as renal agenesis [3].

## Case presentation

A 35-year-old male patient, without any notable medical history, presented at our urological emergency room complaining of pain in the left groin area. The physical examination identified an underdeveloped left testicle, whereas the right testicle was not found in the right hemiscrotum.

**Figure 1:**
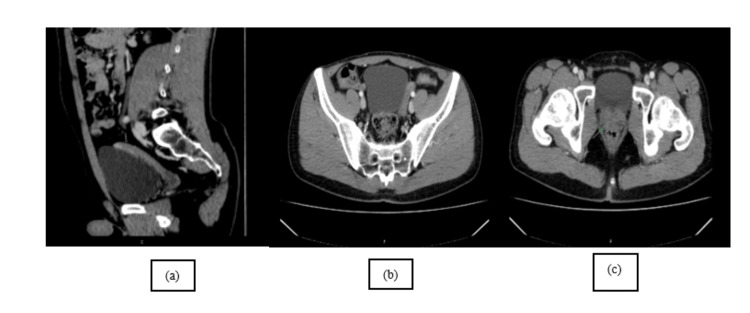
Sagittal venous phase (a) and axial arterial phase (b) CT scans demonstrate a smooth contour, macronodular, with isoattenuation and minimally enhancing ovoidal mass arising from the pelvis, lateral and superior to the bladder. Axial arterial phase (c) CT scan obtained lower demonstrates the presence and normal anatomy of the prostate gland. This figure is the original work of the authors. Patient consent for the use of the image was obtained, as mentioned in the patient consent form.

**Figure 2:**
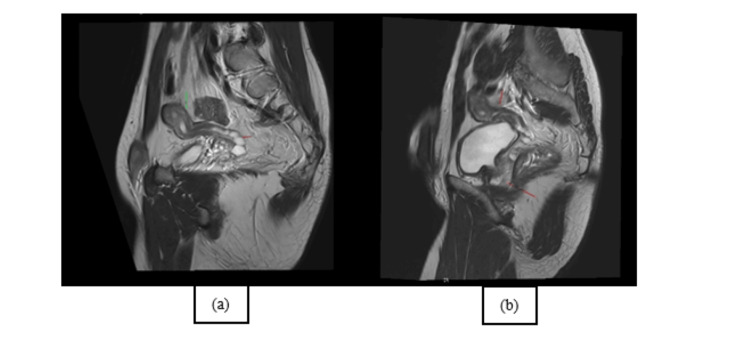
Sagittal T2-weighted image (a) and sagittal oblique T2-weighted image (b) show the zonal anatomy with the endometrium, which is hyperintense; the junctional zone as a hypointense band; and the outer myometrium with an intermediate signal. Also, on the sagittal T2-weighted image (a), there are also multiple cystic-like round nodules compatible with seminal vesicles; the sagittal oblique T2-weighted image (b) demonstrates the normal prostate anatomy shown before on CT scans. This figure is the original work of the authors. Patient consent for the use of the image was obtained, as mentioned in the patient consent form.

**Figure 3:**
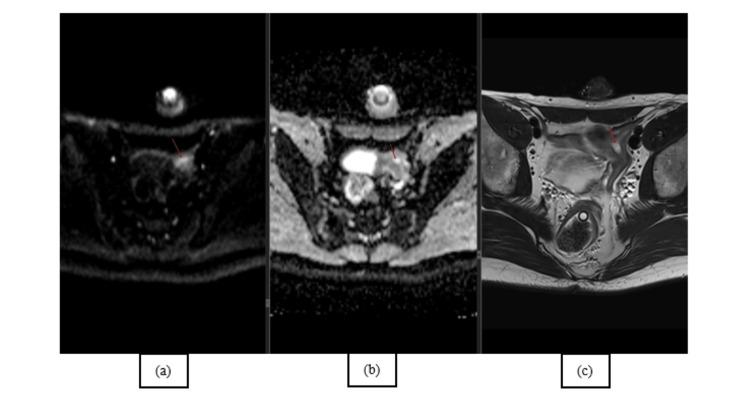
Trace (a) and ADC (b) images show a signal increase in the center of the uterine structure; the axial T2 (c) confirms the T2 shine-through artifact compatible with the endometrium. ADC: apparent diffusion coefficient This figure is the original work of the authors. Patient consent for the use of the image was obtained, as mentioned in the patient consent form.

The CT results showed a clearly defined mass in the pelvic region, situated superior to the bladder having "a uterine disposition" (Figure 1a, 1b, 1c). Even if morphologically descriptive, the body CT scan was unequivocal; therefore, a body MRI was performed, which confirmed Müllerian duct derivatives (i.e., uterus, cervix, and upper third of the vagina) in a close relationship with the prostate and the seminal vesicles. The sagittal T2-weighted image shows the normal anatomy of the uterus with three strata; however, the endometrium drains in the prostatic urethra (Figure 2a, 2b). To further confirm the anatomy, the diffusion-weighted imaging (DWI) and corresponding apparent diffusion coefficient (ADC) map showcase the increase in signal of the endometrium, which is validated on the axial T2 as a T2 "shine-through" artifact (Figure 3a, 3b, 3c).

**Figure 4:**
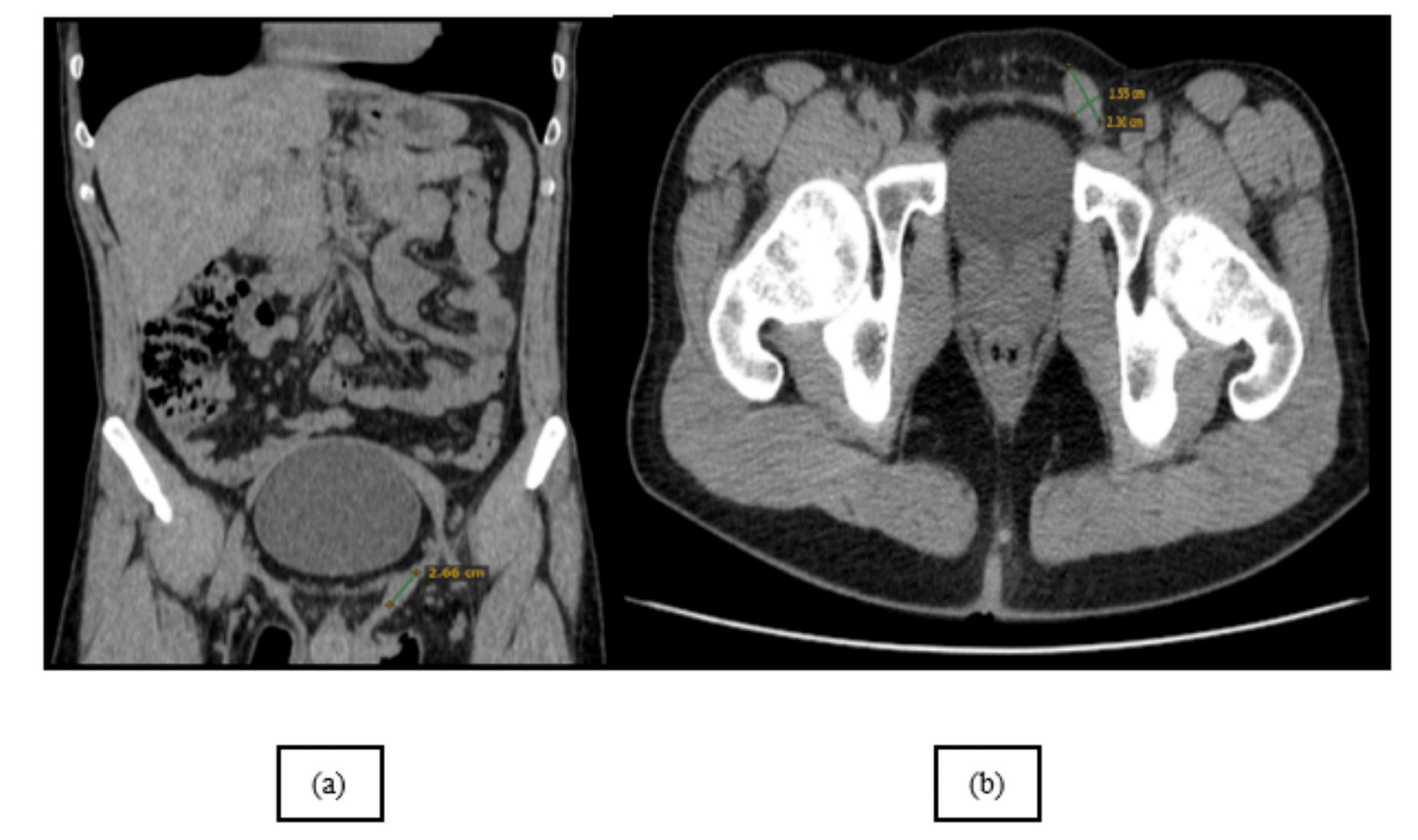
Coronal and axial NECT scans of the pelvis show a small round, isoattenuating mass in the upper part of the left inguinal canal. NECT: non-contrast-enhanced computed tomography This figure is the original work of the authors. Patient consent for the use of the image was obtained, as mentioned in the patient consent form.

**Figure 5:**
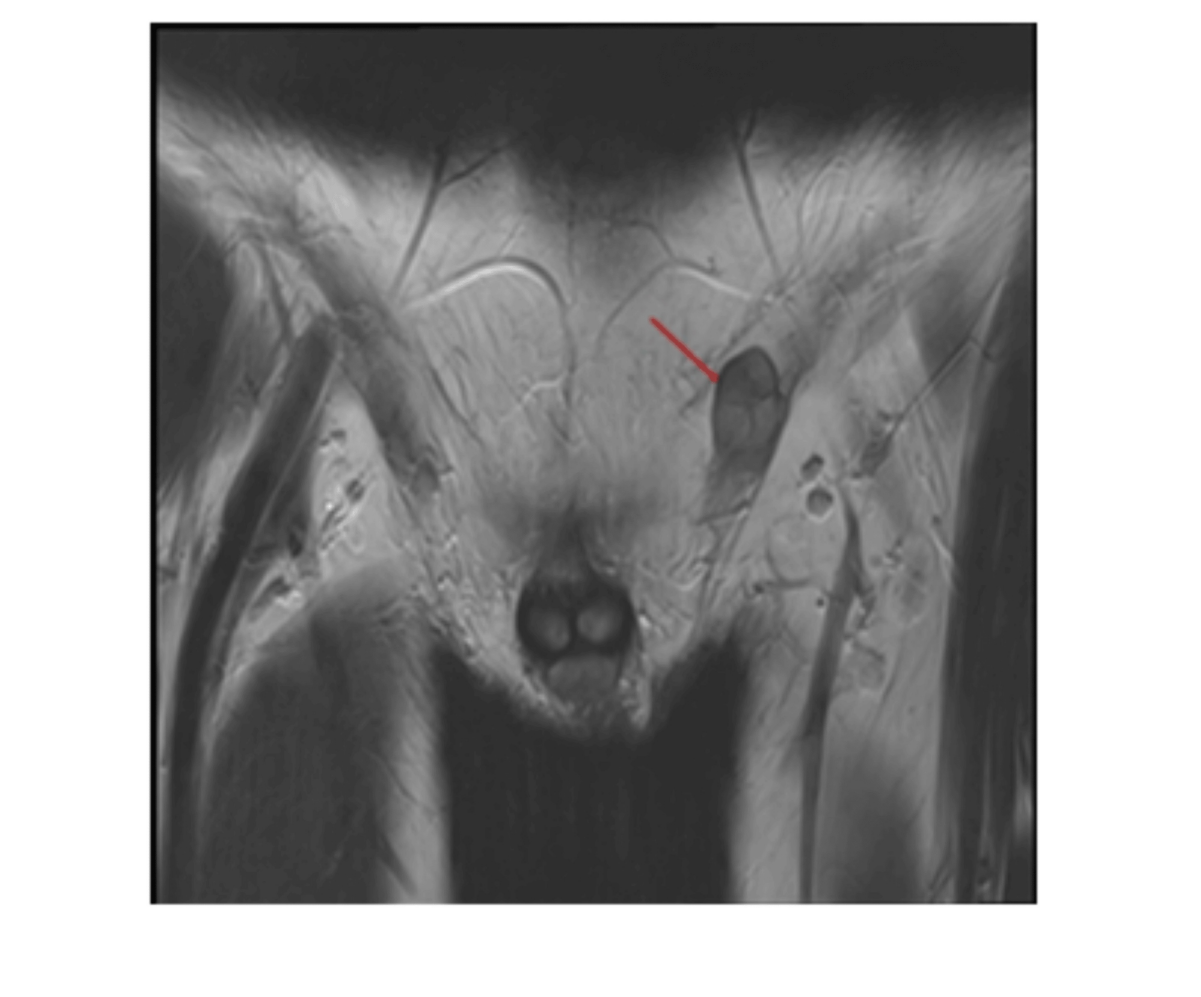
Coronal T2-weighted image MRI scan of the pelvis shows a small heterogeneous mass, with iso- and high T2 signal which raised the suspicion of hypoplastic testicle against Müllerian duct derivatives (further described below). This figure is the original work of the authors. Patient consent for the use of the image was obtained, as mentioned in the patient consent form.

Additionally, there was an oval-shaped mass in the upper portion of the left inguinal canal, which could not be definitively identified on the body CT scan, leading to suspicion of a Müllerian vestige (Figure 4a, 4b). Again, the MRI was diagnostic both with the coronal T2 (Figure 5) showcasing the heterogeneous mass and also with the follow-up sequences. 

**Figure 6:**
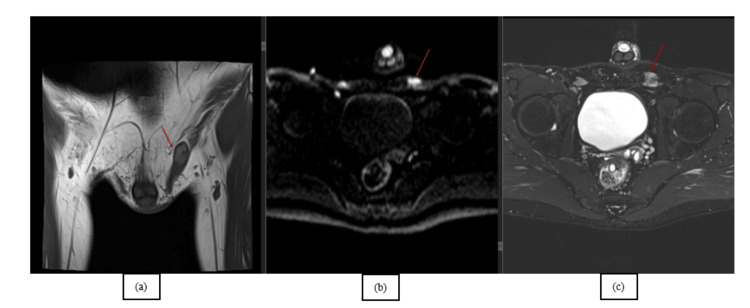
Coronal T1-weighted image (a), trace image (b), and T2FS (c) confirm the diagnosis of cryptorchidism with typical T1 appearance of the undescended testes and the spermatic cord; the high signal on the trace image is fully diagnostic of the testes. This figure is the original work of the authors. Patient consent for the use of the image was obtained, as mentioned in the patient consent form.

Coronal T1-weighted images showcase the undescended testes and the spermatic cord. Furthermore, high signals on the DWI and on the T2FS confirm the diagnosis of cryptorchidism (Figure 6a, 6b, 6c).

Based on the laboratory analyses, the diagnosis of compensated hypergonadotropic hypogonadism could be confirmed. The semen study indicated the absence of sperm, and the genetic examination indicated a normal chromosomal structure. After the cystoscopy, the urethra and prostatic lodge are shown to have a normal appearance. The decision has been made to proceed with the surgical intervention for the pelvic tumor. The pelvic tumor is removed with laparoscopic transperitoneal surgery. An elastic and triangular tumor growth measuring around 6/4/3 cm is observed. It has a bicornuate appearance, with one horn extending towards the left deep inguinal hole and the other towards the right deep inguinal hole.

**Figure 7:**
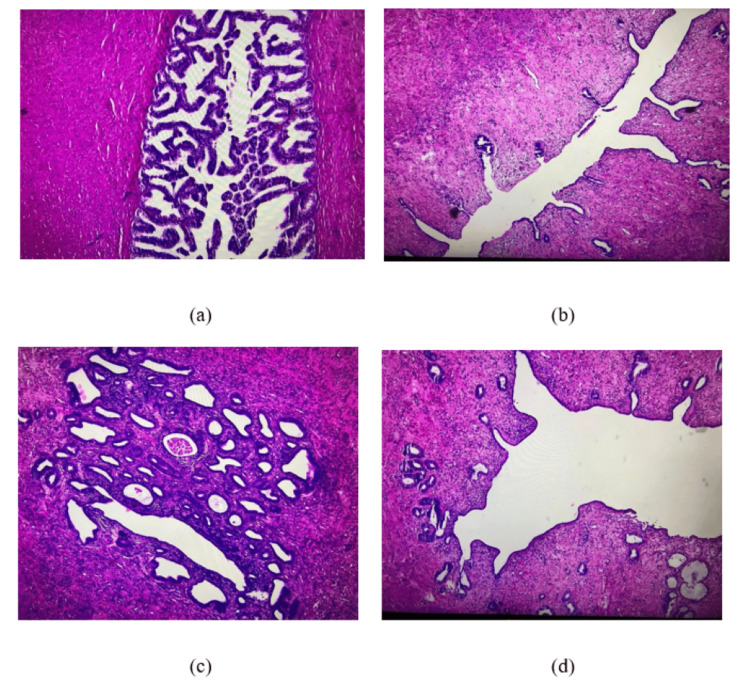
Several microscopic images taken with a 4× objective lens and stained with H&E during the histopathological examination: (a) deferent duct, (b) uterus with endometrial focus, (c) uterus at a different level of sectioning with the endometrium and several grouped glands, some with a dilated appearance, and (d) uterus with slightly dilated uterine cavity and endometrium with a few glands. H&E: hematoxylin and eosin This figure is the original work of the authors. Patient consent for the use of the image was obtained, as mentioned in the patient consent form.

Pathological analysis further revealed a pelvic tumor formation localized to the left lateral vesical region, with a triangular shape measuring 6/4/3 cm, exhibiting a firm-elastic consistency and a bicornuate (uterine-like) appearance. On sectioning, a cavitary appearance is noted with a thickened mucosa (endometrium). Bilaterally along the exterior of the uterus, rigid cords are observed. Microscopically (Figure 7), the uterine wall consists of muscle fibers, small vessels with thickened walls, and endometrium exhibiting simple glandular hyperplasia and edema. Additionally, a small adenomatous polyp is present at the uterine fundus. On either side of the uterus, structures composed of concentric muscle fibers and connective tissue are observed, with a central papillary glandular appearance and the presence of pigment structures compatible with the histological composition of the vas deferens.

The postoperative evolution was favorable, and the patient was discharged with a good general condition, afebrile, undergoing surgical healing. Regrettably, we lost the patient at follow-up as there were no further visits.

## Discussion

A rare form of MPH that is defined by the presence of the uterus, fallopian tubes, and upper part of the vagina in an otherwise normally differentiated 46,XY male is known as PMDS. The syndrome can be attributed to either an inadequate quantity of AMH or an insensitivity of the organ that is the target of the disease to this particular risk factor. Because it is a rare disorder, there are just a few case series that are very small, and there are no prospective studies that can provide clear guidance regarding treatment or long-term management [4-12].

In the published research, there are approximately 200 cases that have been reported. It is typically found in people who are being evaluated for infertility or cryptorchidism or while they are undergoing surgery that is performed on the abdominal region [13,14]. Regarding our situation, the results of the physical examination showed that the patient possesses a typical pattern of external genitalia. In spite of this, the patient was diagnosed with bilateral cryptorchidism and hypoplastic scrotum symptoms. Following the completion of the chromosomal investigation, it was discovered that the male karyotype known as 46,XY was seemingly normal, with no numerical or structural abnormalities.

Despite the fact that some of the individuals had normal spermatogenesis, infertility was a consequence of PMDS that was cited rather frequently. In the case that we examined, it was found that the patient had secondary infertility. When compared to patients who have an isolated undescended testis, patients who have PMDS have a significantly increased risk of acquiring testicular cancer. A total of 20 cases of PMDS with testicular cancer have been reported up to this point [15].

Seminoma is the most rare form of testicular cancer, followed by embryonal carcinoma, teratocarcinoma, and choriocarcinoma. Seminoma is the most common type of testicular cancer. On the other hand, there are findings that indicate that the incidence of Müllerian cancer is significantly lower than that of testicular cancer. Farikullah et al. have reported a significant number of cases of malignant transformation of Müllerian derivatives in patients with PMDS. The rate of malignant transformation ranged from 3.1% to 8.4% [16,17].

In the treatment of patients with PMDS, the primary objectives are to control the undescended testes and Müllerian duct derivatives, to prevent the development of cancer from Müllerian remnants, and to safeguard the patient's fertility.

One of the most notable aspects of our case is the patient's age, as the majority of diagnoses connected with this ailment are made during childhood.

## Conclusions

This paper aims to highlight a unique instance of PMDS and provide a comprehensive review of the existing literature. PMDS should be considered as a potential diagnosis for patients who have either one or both testicles that have not descended. The diagnosis mostly relies on clinical, radiological, or intraoperative findings. The management should focus on eliminating the harmful characteristics of this entity and ensuring the preservation of the patient's ability to reproduce. Surgical removal of the Müllerian duct remnant is recommended.

The primary responsibility of the radiologist is not to identify a specific diagnosis but rather to precisely illustrate the structure of the genitourinary tract and the impact of the ailment on adjacent organs.
